# Sociocultural heterogeneity in a common pool resource dilemma

**DOI:** 10.1371/journal.pone.0210561

**Published:** 2019-01-17

**Authors:** Stefan Gehrig, Achim Schlüter, Peter Hammerstein

**Affiliations:** 1 Department of Social Sciences, Leibniz Centre for Tropical Marine Research (ZMT), Bremen, Germany; 2 Department of Business & Economics, Jacobs University, Bremen, Germany; 3 Institute for Theoretical Biology, Humboldt University, Berlin, Germany; University of Waterloo, CANADA

## Abstract

Collective action of resource users is essential for sustainability. Yet, often user groups are socioculturally heterogeneous, which requires cooperation to be established across salient group boundaries. We explore the effect of this type of heterogeneity on resource extraction in lab-in-the-field Common Pool Resource (CPR) experiments in Zanzibar, Tanzania. We create heterogeneous groups by mixing fishers from two neighbouring fishing villages which have distinct social identities, a history of conflict and diverging resource use practices and institutions. Additionally, we analyse between-village differences in extraction behaviour in the heterogeneous setting to assess if out-group cooperation in a CPR dilemma is associated with a community’s institutional scope in the economic realm (e.g., degree of market integration). We find no aggregate effect of heterogeneity on extraction. However, this is because fishers from the two villages behave differently in the heterogeneity treatment. We find support for the hypothesis that cooperation under sociocultural heterogeneity is higher for fishers from the village with larger institutional scope. In line with this explanation, cooperation under heterogeneity also correlates with a survey measure of individual fishers’ radius of trust. We discuss implications for resource governance and collective action research.

## Introduction

Collective natural resource use and environmental conservation are typically plagued by social dilemmas [1–3]. Yet, contrary to the prediction that resource users should always be incentivised to overexploit common resources [4], convincing empirical evidence has accumulated from case studies all over the world that communal and bottom-up governance are able to support sustainable natural resource use in common property settings [3,5–8]. In small-scale resource systems, local institutions can evolve based on cultural knowledge and habits, shared values and trusting personal relations, which can prevent destruction of the commons by drawing on the social embeddedness of resource users and their potential for cooperation [9–13]. Yet, when ecosystems span beyond human-made social, cultural or political borders, cooperation beyond the immediate community is necessary to sustain its natural resources [1,14]. This raises the question in how far sociocultural heterogeneous stakeholder groups (e.g., with respect to origin, ethnicity, caste or other types of social identity) are able to solve social dilemmas of natural resource governance. Research from diverse case studies shows positive, negative and neutral effects of heterogeneity on collective action for sustainability [15–17]. This is at least partially because diversity affects different aspects of the collective challenge in different ways, so that, in aggregate, “heterogeneity may function as a strength or a weaknesses in conservation problem solving” [1], p. 9). In this paper, we employ an experimental economics approach to investigate how sociocultural heterogeneity affects *cooperativeness* in a common pool resource (CPR) dilemma (research question 1). This excludes other pathways through which heterogeneity may positively (e.g., diversity of knowledge and assets) or negatively (e.g., differing perceptions about resource use) affect sustainable outcomes [17]. For this aim, we conduct lab-in-the-field CPR experiments in Zanzibar involving fishers from two neighbouring villages that have distinct social identities, a history of conflict and diverging resource use practices and institutions. The effect of sociocultural heterogeneity will be discerned by experimentally forming groups that mix fishers from both villages. Lab-in-the-field CPR experiments are a well-established method to assess the role of contextual variables on real resource users’ collective action [8,18–21].

Furthermore, we exploit the fact that the two villages differ in their institutional setup to address whether *institutional scope* can mediate the effect of sociocultural heterogeneity on collective action (research question 2). Following the anthropological literature, we define institutional scope as the extent to which day-to-day social and economic interactions cross-cut group boundaries and extend beyond family relations and small-scale communal networks [22]. Previous literature suggests that institutional scope in the economic realm (i.e. the scale of market and workplace interactions) co-evolves with cultural norms of impartial and generalised, as compared to parochial cooperation [23–30]. This research, however, has rarely been linked to CPRs. Is it conducive to cooperation in a multi-group resource dilemma when local norms cooperation and trust reach beyond the local in-group due to an increased scale of economic exchange? By comparing extraction behaviour in experimentally formed heterogeneous groups between fishers from two villages with different institutional scope, and a survey-measure of radius of trust, we can evaluate this additional hypothesis, albeit only correlational. Put differently, we look at how “culture and context interact” [26], p. 813). Our research thus follows the recommendation that CPR field experiments should be “enriched by collecting […] information about the micro-situational variables and the local social–ecological context of the commons action arena”, for example by “designing samples of several sites or locations” [18], p. 1578) to better explain determinants of collective action.

## Research sites and hypotheses

Our study sites are two fishing villages in the tropical marine inshore fishery of Chwaka Bay, Zanzibar, Tanzania. The Bay comprises a diverse seascape with reefs, seagrass meadows, mangroves and tidal flats and is the main provider of livelihoods for all adjacent villages. The majority of men participate in the multi-gear, multi-species fishery, while many women farm seaweed or collect invertebrates [31]. Poverty remains severe and widespread, and there are indications of over-exploitation of fisheries resources [32–35] while formal resource management institutions are weak [36,37]. The vast majority of the Bay population is Swahili and Muslim with common descent from the Hadimu tribe [38]. The two study villages Chwaka (CH, appr. 3,000 people) and Marumbi (MA, appr. 1,000 people) are characterised by many years of social and political struggle. Despite the geographical proximity of the villages (appr. 4 km) and shared ethnicity and religion, ongoing conflicts have cemented distrust and distinct social and political identities as well as between-village variation in world views, attitudes and cultural-cognitive institutions about how fisheries resources should be used [37,39,40]. This makes the two villages an ideal study site for the effects of sociocultural heterogeneity on cooperativeness in CPR dilemmas. In addition, we can draw on a large body of previous research on the area [41]. Since CH and MA differ in their scope of cooperative economic institutions, these study sites also qualify for an investigation of whether such differences are associated with levels cooperation in a heterogeneous CPR dilemma. This is also relevant for local policy, because, currently, researchers are pessimistic about between-village cooperation for Bay-level fisheries governance [37].

The divergence of characteristics among the two communities has historical roots. CH is the largest, most economically developed and historically most important settlement in the Bay: “[b]y the end of the 18th and early 19th century, Chwaka was one of the most developed villages in Zanzibar, with a vibrant cultural and commercial life” ([42], p. 283). CH was an administrative centre during the times of the British protectorate, and, due to its proximity to the Bay’s mangroves, became a hub of natural resource exploitation and trade as early as the first half of the 20th century [38,43]. This gave rise to new infrastructure (e.g., a road to Zanzibar Town, schools, piped water supply) as well as economic diversification and commercialisation, as apparent in the “replacement to a great extent of a subsistence by a cash economy” ([38], p. 5). In combination with the ingress of “strangers” (ibid, p. 17), this meant to a large degree the “dissolution of the indigenous social structure” (ibid, p. 4) and the dawn of ecological destruction in the Bay. The process of modernisation and development in CH also led to new forms of economic organisation. Service professions like wood traders, fish sellers or bus drivers emerged in the middle of the 20^th^ century [38]. Fishing in large informal “companies” became the norm due to the need for increased manpower after capitalisation of the fisheries by outside investors and the distribution of fishing nets by the government [42]. This led to the replacement of traditional kinship-based production with a modern workplace organisation. Importantly, these transformations remained geographically restricted to CH, creating “conditions which probably apply in as great a degree to few other purely Hadimu communities” [38], p. 33). Still today, CH has one of the largest and most important fish markets on the island [32], with opportunities for Bay trade and rural-urban trade directly at the landing site in the village centre [43]. Also, economic diversification and collaborative production above the household-level are more widespread in CH. For example, net fishers often collaborate in crews of up to 20 unrelated fishers [39]. In MA, in contrast, marketing through long-term relations with middlemen, fishing in small kin-based groups of 2–3 people, and the use of traditional passive fishing gear remain widespread and socially encouraged in MA (to give an example of a typical narrative, one preliminary interviewee from MA expressed that net fishing is a “habit of theirs [fishers from CH]” which “we don’t want here”). Note that the modern, commercially incentivised fishing practices in CH are considered less environmentally sustainable than the use of traditional fishing in MA [34,35,39].

Taken together, this leads to the hypotheses for our two main research questions:

(1) Groups consisting of fishers from both, MA and CH (i.e., socioculturally heterogeneous groups), extract more (i.e., cooperate less) in the CPR experiment than groups with fishers from either only MA or only CH (i.e., socioculturally homogeneous groups).
(2) Fishers from CH (where institutional scope is larger) show less extraction (i.e., more cooperation) in groups consisting of fishers from both, MA and CH, than fishers from MA (i.e., socioculturally homogeneous groups).

Hypothesis (2) is partially based on the idea that social norms of trust differ between the villages with different institutional scope, because trust generalises to a wider social radius when the scale of economic exchanges and social interactions is larger [44–46]. We thus have an additional hypothesis for an effect of trust on the individual level:

(3) Fishers with a larger radius of trust (i.e., less in-group-biased trust) show less extraction (i.e., more cooperation) in groups consisting of fishers from both, MA and CH (i.e., socioculturally homogeneous groups).

Note that our data will also allow us to compare cooperation in homogeneous groups between villages. We do, however, not have an a priori hypothesis about this outcome, and will thus describe and discuss it on exploratory grounds.

## Methods

We followed the usual standards of ethical conduct (no internal Institutional Review Board was in place at the time the study was conceived). All required permits and approvals pertaining to foreign researchers were obtained. In addition, we met with village heads of all involved communities to obtain their verbal approval. All participants took part voluntarily and only after they had given oral informed consent. Written consent could not credibly be obtained from all participants, since some were unable to read. Before giving oral informed consent, participants were informed that (i) even after consent they could opt out from the study at any stage, keeping the money they earned so far, (ii) all of their data was obtained anonymously and (iii) all data would be used for research purposes only. Consent was recorded by asking participants to raise their hands as a sign of approval (S2 Text). All participants approved and stayed until data collection was completed. One participant was underage (16 years). We did not reach out for parental approval, in line with a widely shared principle among ethics committees that young people aged 16–18 with sufficient understanding are able to give full consent to take part in research independently of their parents. We judged his understanding as sufficient. There was no deception in the experiments we conducted and payoffs were distributed privately. Two weeks after finishing data collection, we gave feedback to the communities with preliminary results.

The CPR experiments were presented as a fishing activity. Fishers could choose extraction levels framed as fishing effort and payoffs were reported in gram of fish which, at the end of the game, were converted into local currency. Experiments took place in November 2015 and after one month of preliminary qualitative research by the first author (SG). Subjects could choose fishing effort levels ranging from 1 to 8 over 12 rounds. The constituent payoff function was an n-person common pool dilemma with a concave term for private returns and a linear term for group returns (S1 Text), i.e., the more a person extracted from the common pool, the higher the personal earnings, but the lower the group returns that were shared equally among players (S1 Fig). Fishers played in groups of four. There is only a single subgame-perfect equilibrium for the finitely repeated game (subject did not know the end point, but knew that they would not play more than 20 rounds), which is maximum extraction in every round. The social optimum is at minimum collective extraction (i.e., full cooperation). The equilibrium prediction of full defection (i.e., everyone in the group extracting 8 units) yields 20% social efficiency.

Note that the payoff function differs from other more complex CPR functions in which rivalry of the resource is directly modelled by a proportional individual factor that applies to the benefits from the common pool (e.g., [2]). In line with previous lab-in-the-field experiments (e.g., [47]), we simplified this and payoffs from the common pool-part of the payoff function were distributed symmetrically. Besides making it easier, the symmetric distribution makes the structure of the game strategically equivalent to standard public goods games (see [48]) and our results thus comparable to a broader range of literature on cooperation (which could otherwise be problematic, see [49]).

We use a simple 2 x 2 between-subjects design (Table 1). On one dimension, we vary group composition (socioculturally homogeneous vs. heterogeneous groups), on the other dimension we vary the origin of the participant by sampling from the two different villages. Fishers were sampled with the help of local contact persons who did not know the content of the experiments. Hence, sampling was not fully random. We advised contact persons to publicly invite fishers from the landing sites and to not prefer friends or specific people over others. However, we cannot fully rule out that samples are biased by our sampling method. Fishers who arrived together to the experiment were randomly assigned to experimental groups (two groups per session).

**Table 1:** Treatments and sample sizes in the 2 x 2 design of the CPR experiment. On one dimension, village (CH: Chwaka, MA: Marumbi) is varied by recruiting subjects from two different locations, on the other dimension, sociocultural group heterogeneity is varied by placing subjects either in homogeneous (single-village) or heterogeneous (mixed-village) groups.

	Homogeneity	Heterogeneity
CH	N = 48	N = 20
MA	N = 20	N = 20

The total sample size was N = 108 local male fishers between 16 and 70 years from both villages. Informed consent from participants was elicited during the instructions (S2 Text).

Experiments were conducted in local school buildings. Instructions and all materials were provided in Kiswahili by trained enumerators, who privately assisted subjects that had problems reading and writing. The heterogeneous treatment took place in CH, where we brought subjects from MA via car or local bus. Subjects were seated separately in a classroom, such that they could see whom they were playing with, but not talk to each other or observe decisions. In the heterogeneity treatment, we also mentioned explicitly in the instructions that two players in the group were not local, but from the other village. After each round, subjects got feedback on their payoff and their group’s aggregated extraction (see S2 Text for detailed procedure, instructions and materials). On average, the experiment, including instructions, training exercises and a survey, took 2.5 hours and subjects earned an equivalent of 4.70 USD (SD: 1.02 USD) in cash, which approximates a daily net income from fishing.

Note that the design is unbalanced in two dimensions: Subjects are not evenly distributed among villages (see Table 1) and not all rounds can be used for all subjects. This imbalance resulted from the fact that the sampling scheme and the originally intended treatments had to be adjusted ad hoc in the field (see S3 Text for more details).

Data was analysed in R Version 3.4.3 [50] and all data and code are freely available (S1 File). We ran Tobit panel regressions (censored regression with varying intercepts) using the R packages *plm* [51] and *censReg* [52]. We used Tobit models for the decision data because the dependent variable (extraction) was censored between 1 and 8. The panel data structure results from repeated observations per individual over multiple rounds. We added age, income, wealth and household size to the model as demographic controls. For assessing hypothesis (1), we ran a model with a treatment dummy for heterogeneity only. For hypothesis (2) we added the interaction between heterogeneity and the village a fisher comes from. For evaluating hypothesis (3), we also added a variable that captures the radius of trust, based on the difference in responses to two Likert-type questions in the style of the World Values Survey and its interaction with heterogeneity (taking the difference of trust questions, as in [24], at least partially corrects for different people anchoring themselves differently on the absolute scale and different populations interpreting the wording differently). All self-reported variables were elicited in a private survey that directly followed the CPR experiment (see Table 2 for details and summary statistics and S4 Text for original survey questions). As robustness check, we ran the models also under a mixed-effect ordered logit specification with the R package *ordinal* [53]) with highly similar results (S5 Text).

**Table 2:** Explanation and summary statistics for variables from the survey used in the regressions.

Variable	Explanation	Mean	SD	Min	Max
Age	Age in years	33.5	12.2	16	70
Income	Net daily income from fishing in USD^a^ (average of reports for both monsoon seasons)	7.4	5.0	1.4	32.9
Wealth	First component of Principal Component Analysis on household amenities, correlating with advanced and rare items (e.g., fan, DVD player, smartphone, modern stove)^b^	0	1	-0.8	3.9
Household size	Number of people living in household	5.4	2.2	2	13
Radius of trust	Difference between trust towards strangers and trust towards village members (both measured on a four-point Likert scale)^c^	-1.2	0.9	-3	2

Note: ^a^1 USD equalled 2,130 Tanzanian Shilling at the time of study;

^b^See S6 Text for details;

^c^Variable was constructed such that positive values imply that trust towards strangers exceeds trust towards village members;

## Results

**Fig 1:**
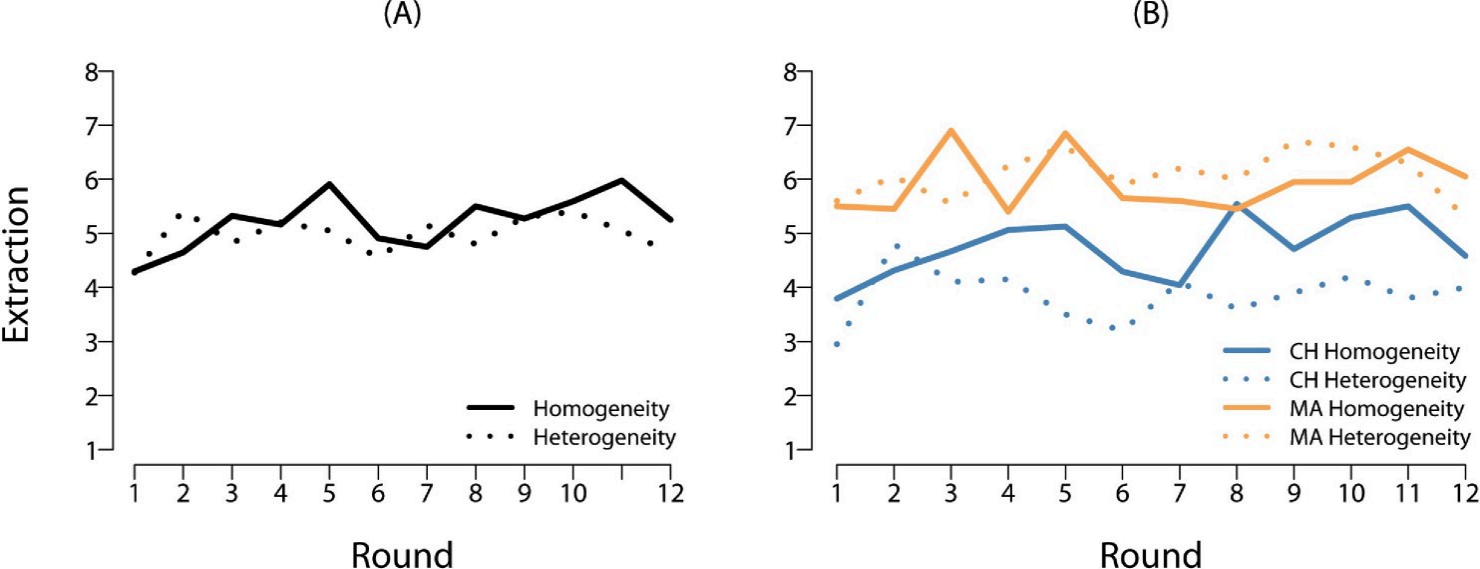
Extraction by round. (A) Mean extraction decisions of fishers under sociocultural homogeneity (groups of fishers from only the own village) and heterogeneity (groups of fishers from own and other village) over time. (B) Mean extraction decisions of fishers from Chwaka village (CH) and Marumbi village (MA) under sociocultural homogeneity and heterogeneity over time.

**Fig 2:**
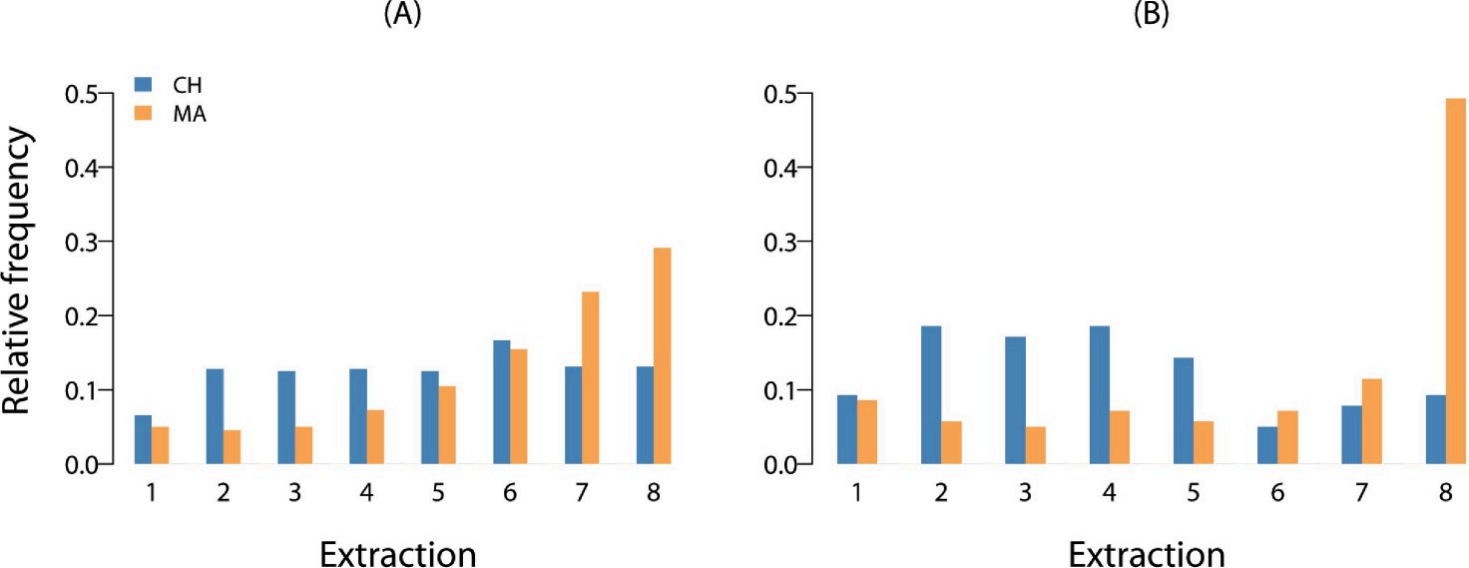
Choice frequencies. Relative frequency of chosen extract levels of fishers from Chwaka village (CH) and Marumbi village (MA) under (A) sociocultural homogeneity and (B) heterogeneity, aggregated over all rounds.

Inspection of the raw data suggests no aggregated treatment effect (Fig 1A), but large village differences in behaviour (Figs 1B and 2).

For a statistical evaluation of our hypotheses, we turn to the results from the Tobit regressions (Table 3). Model 1 shows that, overall, there is no significant effect of sociocultural heterogeneity on CPR extraction (p = 0.67). Thus, the results do not confirm hypothesis (1).

**Table 3:** Tobit panel regressions on CPR experimental behaviour, including demographic (age, income, wealth, household size) and a dynamic controls for round.

	*Dependent variable*:		
	Extraction		
	Model 1	Model 2	Model 3
Heterogeneity	0.17 (0.40)	0.48 (0.54)	-0.52 (0.66)
CH		-1.60^***^ (0.42)	-1.60^***^ (0.41)
Heterogeneity x CH		-1.34^*^ (0.69)	-1.20^*^ (0.64)
Radius of trust			-0.03 (0.22)
Heterogeneity x Radius of trust			-0.90^**^ (0.37)
Age	0.02 (0.02)	0.004 (0.02)	0.003 (0.01)
Income	0.05 (0.04)	0.01 (0.03)	0.001 (0.03)
Wealth	0.09 (0.21)	0.24 (0.19)	0.26 (0.18)
Household size	-0.08 (0.08)	-0.10 (0.09)	-0.10 (0.08)
Round	0.08^***^ (0.03)	0.08^***^ (0.03)	0.08^***^ (0.03)
Constant	4.24^***^ (0.84)	6.32^***^ (0.73)	6.35^***^ (0.76)
Left censored	75	75	75
Right censored	202	202	202
Observations (Subjects)	944 (108)	944 (108)	944 (108)
Log Likelihood	-1,899.47	-1,883.79	-1,880.16
AIC	3,816.94	3,789.59	3,786.33

Note:

^*^p<0.1;

^**^p<0.05;

^***^p<0.01

In Model 2, when the interaction term of sociocultural heterogeneity and village is added to the model to test hypothesis (2), it becomes apparent that, while MA fishers’ extraction in socioculturally heterogeneous groups is increased, CH fishers’ extraction is decreased and, strikingly, even lower than in homogeneous groups (Figs 1B and 2). Although we had no hypothesis about the village main effect (see Methods), we note that fishers from CH extract less in homogeneous groups than their counterparts from MA. Accordingly, mean social efficiency was higher in homogeneous groups from CH (61%) than in those from MA (45%), with social efficiency in heterogeneous groups lying in-between (56%). Taken together, this confirms hypothesis (2) and also demonstrates that the null effect of heterogeneity on extraction identified before results from heterogeneity having inconsistent effects across the two study populations. There is a village difference in the same direction in homogeneous groups (about which we had no a priori expectations), but the village difference in extraction is largest for heterogeneous groups.

In hypothesis (3), we expected that an individual-level measure of in-group-bias in trust explains variation in behavioural differences in heterogeneous settings as well. Indeed, when further adding radius of trust and its interaction with sociocultural heterogeneity to the regressions (Model 3), we find a significant positive association between radius of trust and extraction under heterogeneity, but not under homogeneity. Thus, fishers whose radius of trust is smaller extract more from the common pool, but only in heterogeneous groups. This does not, however, explain all of the village difference in extraction in heterogeneous groups, because adding the trust variable reduces the coefficient for the interaction of heterogeneity only by 0.14 units and the latter remains significant. In all model specifications, there is a small significant time trend with extractions increasing over time.

## Discussion

In this study, we experimentally explored how fishers from a small-scale fishery in Zanzibar cope with sociocultural heterogeneity in a CPR dilemma. Heterogeneity was manipulated by mixing fishers from two villages with a history of social conflict, distinct social identities and diverging cultural traditions of resource use in experimental groups. This had no overall effect on extraction decisions. However, looking at the two communities separately, fishers from the village with larger institutional scope in the economic realm showed more cooperation in the socioculturally heterogeneous setting, as did individuals with a larger radius of trust (i.e., less in-group-biased trust). We discuss these findings in light of the related literature below.

### The effect of heterogeneity

Our finding that, on average, groups comprised of resource users with salient differences in sociocultural group identities show the same level of cooperativeness as those comprised of more homogeneous resource users deviates from much experimental work in behavioural economics and psychology concerned with in-group favouritism (e.g., [54–57]). It is also surprising in the specific context of this study. Social conflicts about natural resource use and politics have been pronounced between the two study villages in the past and have occasionally even led to violent escalations [39,43,58]. Interestingly, yet, the finding is consistent with results from Uruguay, where small-scale fishers did not extract more in mixed-village groups than in single-village groups in a CPR experiment that focussed on punishment [47]. Previous social dilemma experiments not related to natural resource use have similarly found that cooperation in heterogeneous groups (individuals with different ethnicities) can be as high as in homogeneous groups [59,60]. The conclusion we take away from this result is that sociocultural heterogeneity of the type that we investigated does not generally limit cooperativeness in CPR dilemmas.

However, by comparing out-group behaviour across village populations, we could show that this null effect in the aggregated sample is driven by an inconsistent effect on fishers from different villages. This somewhat mirrors empirical work in the CPR literature which emphasises that effects of heterogeneity on collective action are dependent on local institutions and not necessarily negative [17]. We argue that the principal cause for the village difference in out-group behaviour in our setting is the difference in institutional scope [22]. Institutional scope in the economic domain is larger in CH: In contrast to MA, there is a large and important market for rural-urban trade, more developed and diverse businesses and services and fishing “firms”, which can consist of up to 20 people from different households and families on one boat, collaborate in fishing production. In particular the positive effect of team-based workplace organisation on cooperation has previously been shown for fishers [61–63], albeit only in experimental settings that do not explicitly manipulate the social distance between subjects in a group, as we did here. For, example, Carpenter and Seki ([62], p. 614) report higher contributions in a public goods game by those Japanese fishers who pool catches and “exhibit more cooperation in terms of work coordination, effort regulation, and the sharing of information and expertise”. Despite this evidence from other studies, we cannot pin down which village differences exactly cause the observed difference in out-group cooperation. Yet, on a general level, we think that the evolution of large-scale economic exchange in the last century in CH (see [38,42,43,43]) is a convincing candidate for an explanation. Such a transformation is often accompanied by the dissolution of kin and in-group favouritism and the emergence of impartial, more universal norms of cooperation [24,28]. It is in line with the fact that we also found individual radius of trust to be correlated with cooperation in heterogeneous groups, although the scope of trust does not fully explain the village difference.

However, it is surprising that out-group cooperation *exceeds* in-group cooperation in CH (Fig 2), which is a very rare finding in the experimental literature [54]. We point to the interpretation of Schaub [60], who similarly found high experimental cooperation in ethnically heterogeneous groups from neighbouring villages in Georgia and argues that such behaviour could serve to “communicate generally peaceful intentions” (ibid, p. 5), because people “do not want to forfeit the benefits of exchange with their neighbours.” (ibid, p. 6). Psychological research across countries has shown that concerns about group reputation can motivate cooperation with out-group members [56] and anthropologists have pointed out that cultural institutions of inter-group cooperation can evolve when there are incentives for exchange across community boundaries [64]. Such incentives can be larger in interactions with outsiders or strangers than with close peer.

The implication of our experimental results for bottom-up CPR governance is that sociocultural heterogeneity might be less an obstacle for collective action in populations where institutions of generalised trust and large-scale cooperation are more established, even if they have evolved in domains *not* related to resource conservation (e.g., in the economic realm). However, to support sustainability, it is required that institutions develop in such a way that cooperative norms support resource governance and not just exploitation and commercialisation of the fisheries [65].

### Village differences in homogeneous groups: Implications for the external validity of CPR experiments?

We did not have an a priori hypothesis on a village difference in sociocultural homogeneous groups (i.e., single-village groups), but we observe that, as in heterogeneous groups, fishers from CH extract less than fishers from MA. Ex post, this can be aligned with the explanations provided above: Economic evolution might also favour higher levels of cooperation *within* communities. Khadjavi et al. [66], for example, found higher levels of intra-village cooperation in Prisoner’s Dilemma games by those Zambian farmers who had more contact to modern commercial large-scale farms.

Note that our results have implications for the debate on external validity of social dilemma experiments for predicting sustainable behaviour in the real world (see [67–72]). We found more cooperation in a CPR game in the community for which less sustainable fishing practices are reported [34,35,37,39]. If this is, as in our interpretation, due to economic (rather than environmental) institutions of cooperation, these contextual factors could be a confound when researchers try to measure sustainable behaviour with social dilemma experiments across populations, as has been common in the past.

Of course, the village-level divergence between real-life sustainability and cooperation in the CPR game could also stem from the fact that there is indeed a stronger norm or preference for sustainability in CH, but, in their day-to-day lives, fishers in the community are constrained to choosing destructive fishing gear due to lack of knowledge or capital (see [73]). To assess this, we exploit further survey questions asked to the same participants (for a more detailed analysis of these questions, see [40]) in additional regressions (S7 Text). Proxies for concerns about both general sustainability and the destructiveness of one’s own fishing technique do not show significant associations with extraction decisions and do not affect the village-level estimates. This gives confidence that the cooperativeness we measure in the CPR games is not strongly related to environmental concern, but to another factor.

Consequently, we believe that experimenters should more clearly distinguish populations of resource users which actively engage in community-based or co-management of resources from those that do not when arguing for or against external validity. Studies with forest users in Ethiopia [70] and fishers in Chile [68] and Mexico [74] show more experimental cooperation among groups that are more actively involved in resource management. The fishers we recruited, in contrast, have little experience in sound community-based governance [37]. Thus, external validity of CPR experiments for predicting sustainable behaviour might depend on the most salient domain of cooperation that subjects perceive the game to be about [75], even independently of whether the experiment is framed or not (see [76]). If people, in line with the actual payoff structure, recognise the game as economic exchange rather than resource conservation, environmentally exploitative groups could be observed to cooperate well in CPR experiments (remember the strategic equivalence of our CPR game with a public goods game, see Methods). This perspective on external validity is a fruitful avenue for future studies and also points to a more general observation on the role of cooperation in sustainable development, which we develop below.

### Does rapid economic development spur cooperation without sustainability?

Ecological degradation and increasing institutional scope of cooperation might co-evolve under rapid economic development. This is because development and market integration typically create incentives for both, increasingly impersonal cooperation in the domain of economic transactions like trade and production, but also the over-exploitation of natural resources due to access to new markets and harvesting technologies [77]. This underlines that cooperation per se is not enough for sustainability, because, more generally, “individuals with common interests in one form of cooperation may have no interests at all in cooperating to advance other collective interests” ([78], p. 112). Institutional change in the domain of sustainability needs to follow these economic transformations to overcome the typically observed “valley” of low sustainability in areas that undergo rapid growth and development [79]. For example, appropriate policies could make CPR cooperation more akin to other daily social dilemmas that people are habituated to solve in the economic domain, e.g., by making penalties and rewards more immediate (i.e., set present-day incentives through monitoring), decreasing costs of cooperative restraint from extraction (i.e., decrease resource dependence), reducing group size (i.e., limit access to the system) or providing more certainty about the payoff structure of a CPR scenario (i.e., raise environmental awareness and education). These recommendations overlap with popular concepts for the solution of commons dilemmas [1,3,65].

### Limitations, alternative explanations and outlook

Our study has obvious limitations. Conclusions are based on data from a rather small sample of participants, as typical for lab-in-the-field experiments that focus on rural communities (e.g., N = 44 [47], N = 56 [22]). Also, our sampling procedure was not fully randomised and, by design, we excluded women as participants, because we wanted to focus on fishers’ behaviour. However, women's activities in Chwaka Bay [80], as in all small-scale fisheries, are economically and ecologically significant.

Further, although we identify the hypothesised village difference in CPR extraction behaviour in heterogeneous groups (hypotheses 2), any causal attribution remains speculative (i.e., that part of our study is correlational), despite being backed up by a careful examination of the local context. Moreover, as typical for case studies, we provide only two data points on the community level. Alternative explanations, not based on institutional scope and associated cultural norms, for the result that fishers from CH village acted more cooperatively than their counterparts from MA (in both, mixed-village and single-village groups), remain. Below, we discuss some of them.

First, it is possible that the population difference is the result of mere migration driven by individual preferences. Yet, this is unlikely, as only 15 out of the 108 fishers in the sample reported to have moved to another community in their lifetime and the majority of those did so as children.

Second, a concern might be that CH, by being larger and more politically important, had greater exposure to formal development programs and initiatives by outside organisations, including those targeting cooperation over sustainable fisheries management and the resolution of the conflict between MA and CH. Such exposure might trigger strategic considerations among subjects attempting to signal ethical convictions that could result in future money from development programs. The reported village differences in CPR extractions might then, at least to some degree, be an artefact resulting from behaviour being observed by the researchers. We cannot thoroughly address this concern, but we can at least show that samples from CH and MA, based on self-reports in the survey, did not differ in the strategic goals they pursued during the experiment (S8 Text). However, it is important to keep experimenter demand effects in mind when interpreting behavioural field experiments, especially in remote locations [81].

Third, behavioural differences among villages might be due to ecological, rather than cultural factors (e.g., [82]). We expect that most ecological conditions like the type of fishing habitat and biophysical conditions are rather similar between the nearby villages. Yet, historically, a strategic location and the proximity to mangrove forests could have been an important reason for CH becoming a commercial and political hub in the first place [38]. The novel socioecological conditions that resulted from this and changed the incentives for large-scale cooperation (e.g., markets, strangers, specialisation) do, however, not speak against a cultural explanation. Socioecological factors can govern the evolution of culture [83].

It has become clear in previous studies that sociocultural heterogeneity of resource users can have diverse effects on sustainability outcomes [15–17]. In this study, we showed that the effect heterogeneity has on pure *cooperativeness* (ignoring other consequences of resource user heterogeneity in CPR scenarios) can be inconsistent across two communities from the same social-ecological system. In general, this encourages more research on drivers of cooperation on small scales, i.e., on those drivers that create variation between villages of the same ethnicity [82] or between neighbourhoods of the same city [84]. Future research should assess the effect of heterogeneity on CPR harvest in more diverse contexts and try to more precisely pin down which institutional factors mediate this effect. Further, our results suggest that sustainable behaviour should be understood as one of many domains and levels of cooperation that people and communities engage in, and not, as other’s have argued [67], as a context-independent preference. Appropriate methodologies will thus combine lab-in-the-field experiments with more naturalistic measures of behaviour. Research that investigates natural resource governance in a multi-level framework [65,85], and, ideally, also longitudinally [86] will advance our understanding of the evolution of norms of sustainability and cooperation within and across groups.

## Supporting information

S1 Fig Plot of payoff function. (PNG)

S1 File Data and code. (ZIP)

S1 Text Payoff function in the CPR experiment. (DOCX)

S2 Text Details on procedure, instructions and materials. (DOCX)

S3 Text Discussion of unbalanced sample. (DOCX)

S4 Text Original survey questions. (DOCX)

S5 Text Mixed-effect ordered logit specification of regressions. (DOCX)

S6 Text Principal Component Analysis for wealth. (DOCX)

S7 Text Additional Tobit panel regressions. (DOCX)

S8 Text Self-reported strategic goals. (DOCX)
