# Supplementary figures and images for: Sociocultural heterogeneity in a common pool resource dilemma
Source: PLoS One. 2019 Jan 17;14(1):e0210561. doi: 10.1371/journal.pone.0210561 (PMC6336341; doi:10.1371/journal.pone.0210561)

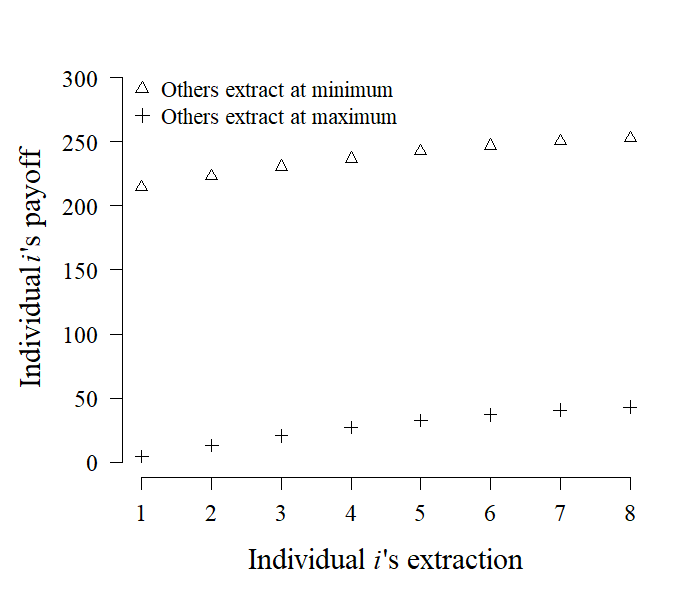

Supplement: S1 Fig — (PNG) [file pone.0210561.s001.png]
