# Supplementary material for: Sociocultural heterogeneity in a common pool resource dilemma
Source: PLoS One. 2019 Jan 17;14(1):e0210561. doi: 10.1371/journal.pone.0210561 (PMC6336341; doi:10.1371/journal.pone.0210561)
Supplement: S1 Text — (DOCX) [file pone.0210561.s003.docx]

**S1 Text. Payoff function in the CPR experiment**

The basic payoff function resembled an n-person social dilemma with a concave term for private returns and a linear term for group returns:

$\Pi_{i}=20x_{i}-\frac{1}{2}x_{i}^{2}+\frac{F_{0}-c\sum_{i=1}^{n} x_{i}}{n};$ (1)

In equation (1), $\Pi_{i}$ represents the payoff for player $i$, $x_{i}$ is the level of, $n$ the number of players, $c$ the social cost factor of the aggregated group extraction, and *F_0_* the initial size of the resource pool that players extract from.

The first order condition for maximizing individual payoff is:

$\frac{{d\Pi}_{i}}{dx_{i}}=20-x_{i}- \frac{c}{n}=0;$ (2)

Setting *n* = 4 for 4 players in a group, equation (2) requires for the payoff-maximizing extraction effort *x_i_** to be:

${x_{i}}^{*}=20-\frac{c}{4};$ (3)

For the parameters chosen in our game, *F_0_* = 940 and *c* = 40, the Nash strategy is the corner solution, i.e. *x_i_** = 8 (the second order condition $\frac{{d^{2}\Pi}_{i}}{d^{2}x_{i}}<0$ is met).

For finding the socially optimal extraction strategy, one has to maximize the aggregated harvest *Π_all_*:

Max $\Pi_{all}=20\sum_{i=1}^{n} x_{i}-\frac{1}{2}\sum_{i=1}^{n} x_{i}^{2}+F_{0}-c\sum_{i=1}^{n} x_{i}$; (4)

For *n* = 4, the first order condition $\frac{{d\Pi}_{all}}{dx_{i}}=0$ requires for the socially optimal effort ${x_{i}}^{so}$:

${x_{i}}^{so}=20-c$; (5)

With our parameter settings, this makes the socially most efficient strategy to be the minimum effort (the second order condition $\frac{{d^{2}\Pi}_{all}}{d^{2}x_{i}}<0$ is met).
