# Supplementary material for: Sociocultural heterogeneity in a common pool resource dilemma
Source: PLoS One. 2019 Jan 17;14(1):e0210561. doi: 10.1371/journal.pone.0210561 (PMC6336341; doi:10.1371/journal.pone.0210561)

**S2 Text. Details on procedure, instructions and materials**

*Procedure*

Local contact persons (a beach recorder in CH and a well-connected fisher in MA, who did not know the purpose of the study and were compensated for their efforts) approached fishers at the landing site, market or village centre the day before experimental sessions and asked them whether they wanted to participate in a research activity the next day, in which they could earn money by making decisions. Fishers who accepted were invited into sessions for the next day. Sessions were scheduled to allow for payers and fishing, with the earliest starting at 9 am and the latest starting at 4:30 pm. The time from people arriving at the schools to receiving their payments always lay between two and three hours. At the beginning of a session, eight subjects were randomly assigned to two groups. We held a maximum of three sessions per day, with always two groups playing simultaneously in adjacent, separated rooms of local school buildings. We paid the schools as compensation for using the rooms and sessions took place outside school hours. Data was collected within six days in November 2015 while the first author was staying in MA and CH (total stay in the study area: 3 months). Four assistants per session helped with administering the games with always two assisting one group. Prior to the experiments, assistants had been trained at the Institute of Marine Science in Zanzibar Town for several days by SG and AS, with the help of local researcher Dr Narriman Jiddawi. Two of them had prior experience in administering economic field experiments. Fishers from MA who participated in the outgroup treatments were either offered to be picked up by car to be brought to CH village, or were refunded the money for a local bus, if available. After the experiments, they were immediately brought back to MA.

The sessions always followed the same structure. After a brief welcome and the handing out of ID numbers to all participants of a session, we divided fishers into two groups, one of which was subsequently led to another room. In the rooms, participants were seated at four separate tables in each corner. Instructions included a verbal and visual description of the game (Supplementary Information 3), as well as quizzes and a test round, which everybody had to answer correctly. Instructions took around 45 minutes. Participants could ask questions at any time and received personal assistance and explanations when something remained unclear. Only after we were convinced that everybody had understood the game, we started. People who couldn’t read or write received additional personal assistance in each round and were explained how to use the colors and directions of the payoff table instead of the numbers to guide decision-making. After each round, assistants collected the decision sheets and SG filled in the feedback information (own payff, others’ aggregated extraction), before they were handed back. The twelve rounds of play took around 30-45 minutes. After the 12^th^ round, it was announced that the game was over, survey sheets were distributed and assistants helped in filling them out in private, one after another. This took around 45-60 minutes. Subjects who had completed the survey received their payment in a closed envelope and could leave the building.

*Instructions*

All instructions and materials had been translated and printed in Kiswahili prior to the experiments. Below, we provide the template that was read out by assistants at start of each session:

Welcome to our fishing game and thank you for taking your time! You can earn money in this activity and we hope it will be entertaining for you. The whole activity will take approximately 2-3 hours. It is made sure that anyone of you earns at least around 4.000 TZS in cash, but you can go home with a lot more, depending on the decisions of yourself and others in the game. You cannot lose any money that you earned. You will be paid after the activity and you receive all the earnings that you achieved during the game.

Your names are not recorded and any information you provide is anonymous and only used for the purpose of our study. We do not record your name and we have no not share any data about you with political officials. You are only allowed to take part in the experiment once. We play different games in your community, so if you heard something about the games from others, you should not base your decisions on what they told you. We will present and explain the results of the games in your community in December, where you can take part in discussions about the game and Chwaka Bay’s fisheries in general.

If you agree with what we just told you and want to take part in the activity, please raise your hand. Otherwise, feel free to ask further questions, or to leave. You can also leave later during the activity at any stage and keep the money that you earned so far.

So let’s get started.

In the game that you will, you will be in a situation in which a group of fishermen uses their resource together, a stock of fish. We do this to get a better idea of how groups of fishermen from Chwaka Bay use the fish stocks.

You have been randomly assigned to a group of 4 players, which are from your community [alternatively: consist of 2 persons from Chwaka and 2 persons from Marumbi; outgroup treatment]. Although you can see who is in your group, you are not allowed to talk to anybody during the whole game. This is why we please you to stay quiet during the game and not make comments or statements to other players about your or their decisions. In case some of you start chatting with each other during the game, we will unfortunately have to stop the game.

So what is the game all about?

In the game, all 4 players of a group go fishing on a common fish stock. When you go fishing on that stock, you can decide on how much effort you want to spend to catch fish. You will earn real money from what you have caught. However, remember that the other fishermen in your group also fish from your fish stock. This means, the yield of fish that you can make in this game is dependent on your own decision about how much you want to fish, but also on the decisions of the others of your group of how much they want to fish. This is natural when many people fish from one population of fish, as you may know it from fishing in Chwaka Bay. Fish harvested by one fisher are not available for other fishers. How much money you earn in the game is determined by your decisions in the fishing game and the total yield you make, so take your time and think carefully before making your choices! Every decision you take during the game will be private and anonymous. The game consists of a series of decision rounds, where you can make a fishing decision in every round. To decide about your decision you have to look at the yield table.

At this point, it is important that you let us know when somebody has difficulties reading or writing numbers. An assistant will help you in that case.

[Yield table is handed out]

How to use the yield table?

This is they yield table, which contains all information you need to make your decisions in the game. In the table, you see how many grams of fish you earn on one fishing day, depending on your and your group’s decisions of how much to fish. One fishing day corresponds to one round of the game. You can exchange the earned grams of fish from all your fishing days for cash after all rounds are finished at a price of 7.000 TZS per kg. When you play a round of the game, you decide how much effort to spend for fishing on a day. You can choose from 1 to 8, which is represented by the columns from 1 to 8 in the table. The higher the number, the harder you try to catch fish. 1 is the minimum amount of fishing in this game for a single round, and 8 is the maximum amount of fishing. You decide for any effort of fishing from 1 to 8. Keep in mind that your decision is absolutely private and the other members of your group will not know any of the decisions that you take. Only the assistants will see the decisions to calculate your earnings, and they not know you personally or take down your name. You will play several rounds, before, at some point, we will tell you that the game is over.

Again, it is important to see that how much you earn depends not only on your own decision, but also on the fishing effort of the others in your group. The fishing effort of the others in your group corresponds to the lines of the table. So, to read your earning from a fishing day, you have to combine the column of your own fishing effort and the line of the total fishing effort of the others in your group.

We added colors to the yield table to make it easy for you to identify high, medium and low yield of fish. We put in green the numbers greater than 200 gram, in yellow the numbers from 100-200, and in red the number of grams smaller than 100.

So, for example, if you go fishing with 1 unit effort, and all the others in your group jointly go fishing at a level of 12 units, you make […] gram of fish on that fishing day. Imagine, on the next day you go for 5 units instead, but the others stay at 12, you will make […] gram. Now imagine, you stay at 5, but the others increase to 22, then you make […] gram of fish.

Are there any questions on how to read the table?

To play the game and make the decisions, you will use the decision form. At first, the player number you received from us must be written on the sheet.

[Decision form is handed out]

How to use the decision form?

You make your decision by filling a number from 1 to 8 in the first column, according to the fishing effort you want to spend on that day. The assistants will collect the forms when all decisions are made at the end of one round. Look at them, and fill in how much fishing effort the others in your group have spent at that fishing day and how much amount of fish you have personally earned. The assistants will write this information in the corresponding columns of your decision form. Then, the next round starts and you can take a new personal decision on how much you want to go fishing this time.

Do you have any questions so far?

As you can see in the payoff table, you get more when you fish more. However, when you look at the upper left corner, you see that it’s the best for the whole group when you all spend only 1 unit of fishing effort. If everyone of you gets 215 gram, as it is shown in the corner, it is the most the whole group can earn. Instead, of you all spend the maximum 8 units of effort, you will only get 43 gram each. But when you look at the numbers from the left to the right, you also see that for higher fishing effort you always get more, while decreasing what the others get.

At some point the game will end, and all your earnings from all rounds will be summed up and multiplied with 7.000 TZS per kg fish. You do not know when exactly it will end, but you will not play more than 20 rounds. After completing the questionnaire with an assistant, you will receive the money for your earnings of fish privately in cash in an envelope.

So let‘s play a quiz and a practice round, to make sure you understand the game. Don’t forget to ask questions when something is unclear. Only when everybody has understood the game, we can start the game.

The quiz is: Imagine you go fishing as little as you can, and each other person in your group of 4 people goes fishing with an effort of 2. How many grams of fish would you earn? Please write the answer in your decision form in the quiz field. The assistants will have a look at it.

We do another quiz: Imagine you go fishing with an effort of 7, and the other three group members go fishing with an effort of 1. How many grams of fish would you earn? Think about the answer. The assistants will come to you, ask what you think, and help you.

Now we play a practice round which will not contribute to your earnings.”

*Materials*

Yield table:


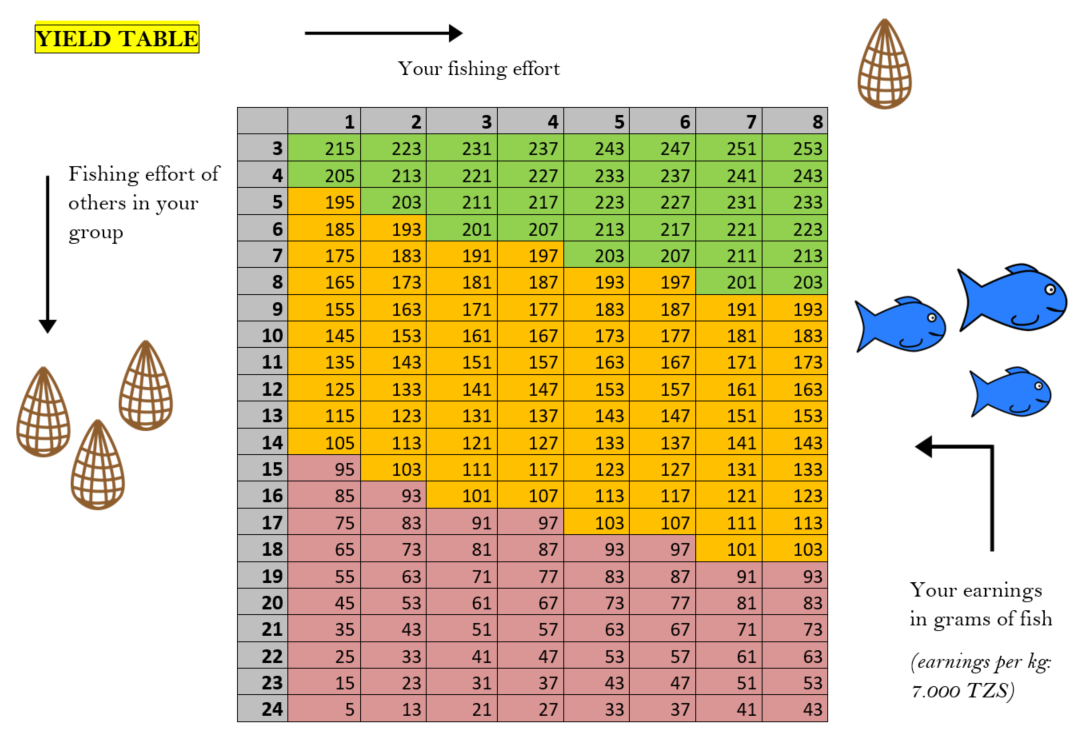


Decision form:


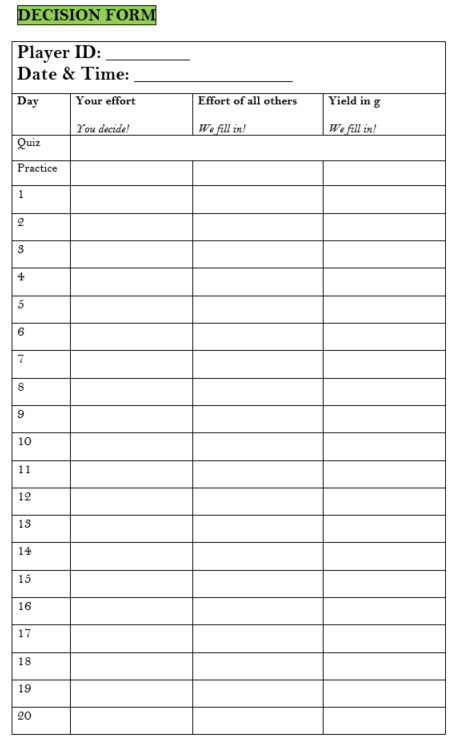

Supplement: S2 Text — (DOCX) [file pone.0210561.s004.docx]
