# Supplementary material for: Sociocultural heterogeneity in a common pool resource dilemma
Source: PLoS One. 2019 Jan 17;14(1):e0210561. doi: 10.1371/journal.pone.0210561 (PMC6336341; doi:10.1371/journal.pone.0210561)
Supplement: S3 Text — (DOCX) [file pone.0210561.s005.docx]

**S3 Text. Discussion of unbalanced sample**

Due to constraints we encountered during field work, the sample we use in this paper is unbalanced in two dimensions. First, there are more fishers from CH village under homogeneous group composition (N = 48) than in all other treatments (N = 20; see Table 1 in the main text). Second, for 44 out of the 108 subjects, only the first four rounds of the CPR experiments can be included in the analysis.

The reason for both is that we had initially planned to run more treatments, including (i) a communication treatment in both, single-village and mixed-village groups (extending on work by, e.g., Burns and Keswell, 2015; Cardenas, 2003, and (ii) treatments with altered payoff functions resembling natural resource scarcity (extending on work by, e.g., Blanco et al., 2015; Cerutti, 2017; Osés-Eraso and Viladrich-Grau, 2007, on effects of the size of the common pool). After the research team had started to run sessions of all these treatments, it was noted that, for future sessions, much less subjects than expected were available in the study area. We were able to sample in total 172 fishers from both villages. We used survey data from this full sample for a different research question (Gehrig et al., 2018). Unfortunately, we had to completely discard the experimental decision data from 64 fishers which were assigned to treatments with different payoff functions. These treatments could not be completed. To maintain statistical power at least for the research questions explored in this article, we decided post hoc to pool fishers from communication treatments and from non-communication treatments (which both had played under identical payoff functions), but only using the first four rounds from the former. This is because communication only started after round four in the communication sessions. Due to this, the sample is unbalanced with respect to rounds. When we had to stop sampling due to budget constraints, less sessions with the appropriate payoff function had been run in MA village, which is why the sample is unbalanced with respect to village.

Importantly for inference, the process that created missing observations in rounds five to twelve for some subjects is rather random (assignment to communication vs. non-communication treatment), as is the process that created unbalance with respect to sample size per village (the order of sessions and locations was pre-defined by the researchers). Further, our analytical approach based on regressions can handle unbalanced samples.

*References*

Blanco, E., Lopez, M.C., Villamayor-Tomas, S., 2015. Exogenous degradation in the commons: Field experimental evidence. Ecological Economics 120, 430–439. https://doi.org/10.1016/j.ecolecon.2015.03.028

Burns, J., Keswell, M., 2015. Diversity and the provision of public goods: Experimental evidence from South Africa. Journal of Economic Behavior & Organization 118, 110–122.

Cardenas, J.-C., 2003. Real wealth and experimental cooperation: experiments in the field lab. Journal of Development Economics 70, 263–289. https://doi.org/10.1016/S0304-3878(02)00098-6

Cerutti, N., 2017. Resource Changes and Cooperative Behavior (PhD Thesis). IRC-Library, Information Resource Center der Jacobs University Bremen.

Gehrig, S., Schlüter, A., Jiddawi, N.S., 2018. Overlapping identities: The role of village and occupational group for small-scale fishers’ perceptions on environment and governance. Marine Policy 96, 100–110. https://doi.org/https://doi.org/10.1016/j.marpol.2018.06.017

Osés-Eraso, N., Viladrich-Grau, M., 2007. Appropriation and concern for resource scarcity in the commons: An experimental study. Ecological Economics 63, 435–445. https://doi.org/10.1016/j.ecolecon.2006.11.016
