# Supplementary material for: Sociocultural heterogeneity in a common pool resource dilemma
Source: PLoS One. 2019 Jan 17;14(1):e0210561. doi: 10.1371/journal.pone.0210561 (PMC6336341; doi:10.1371/journal.pone.0210561)
Supplement: S4 Text — (DOCX) [file pone.0210561.s006.docx]

**S4 Text. Original survey questions**

In which village do you live?

[] Marumbi [] Chwaka

How old are you?

_____ years

What is your average daily income on a normal day?

season: _____ off-season: _____

How many people live in your household?

Total: _____

Which of the following items can be found in your household?

electricity [] present [] absent

fan [] present [] absent

sitting toilet [] present [] absent

radio [] present [] absent

TV [] present [] absent

satellite dish [] present [] absent

VCR/DVD [] present [] absent

Pay-TV [] present [] absent

fridge [] present [] absent

smart phone [] present [] absent

cupboard [] present [] absent

chairs [] present [] absent

modern stove [] present [] absent

cement walls [] present [] absent

cement floor [] present [] absent

metal roof [] present [] absent

motorcycle [] present [] absent

car [] present [] absent

How much do you trust people from the following groups? Completely (1), somewhat (2), not very much (3) or not at all (4)

People from your village [] 1 [] 2 [] 3 [] 4

People you meet for the first time [] 1 [] 2 [] 3 [] 4

Imagine you couldn’t fish in Chwaka Bay anymore, what is a realistic alternative for you to make a living?

[] making income with ___________________

[] relying on income from family or friends

[] moving away from Chwaka Bay

[] I don’t see possible alternatives

For how many years have you been living in your village?

[] all my life [] _____ years

What was your goal during the game?

[] earn as much as possible

[] earn more than the others

[] earn as much as the others

[] make the others earn as much as possible

[] no specific strategic goal

Are you worried when you think your children will have to use the same fishing grounds as you do?

[] yes [] no

What are the main fishing techniques you use? (Select 1 or 2! If 2: give ranks 1 and 2 regarding frequency)

[]__ dragnet []__ basket trap

[]__ purse seine net []__ fence trap

[]__ spear []__ gill net

[]__ handline []__ speargun

[]__ other: ______

Which of the fishing gears do you find most destructive and harmful in Chwaka Bay?

[] dragnet [] basket trap

[] purse seine net [] fence trap

[] spear [] gill net

[] handline [] speargun

[] other: ______ [] none
