# Supplementary material for: Sociocultural heterogeneity in a common pool resource dilemma
Source: PLoS One. 2019 Jan 17;14(1):e0210561. doi: 10.1371/journal.pone.0210561 (PMC6336341; doi:10.1371/journal.pone.0210561)
Supplement: S5 Text — (DOCX) [file pone.0210561.s007.docx]

**S5 Text. Mixed-effect ordered logit specification of regressions**

The three columns show the models from Table 3 in the main text in the same order, but for a mixed-effect ordered logit specification. Individual subject is the random effect. Constants (threshold coefficients for all extraction levels) are not reported.

|  | |  |  |
| --- | --- | --- | --- |
|  | *Dependent variable:* | |  |
|  |  |  |  |
|  | Extraction | | |
|  | (1) | (2) | (3) |
| Heterogeneity | 0.10 (0.28) | 0.32 (0.37) | -0.25 (0.48) |
| CH |  | -1.08^***^ (0.31) | -1.06^***^ (0.29) |
| Heterogeneity x CH |  | -0.89^*^ (0.49) | -0.85^*^ (0.47) |
| Radius of trust |  |  | 0.05 (0.15) |
| Heterogeneity x Radius of trust |  |  | 0.55^**^ (0.27) |
| Age | 0.007 (0.01) | 0.003 (0.01) | 0.002 (0.01) |
| Income | 0.03 (0.03) | 0.002 (0.02) | -0.002 (0.02) |
| Wealth | 0.10 (0.15) | 0.17 (0.13) | 0.17 (0.12) |
| Household size | -0.10 (0.07) | -0.07 (0.06) | -0.07 (0.05) |
| Round | 0.05^***^ (0.02) | 0.05^**^ (0.02) | 0.05^***^ |
|  | |  |  |
| Std. Dev. Random Effect | 1.19 | 0.96 | 0.89 |
| Observations (Subjects) | 944 (108) | 944 (108) | 944 (108) |
| Log Likelihood | -1817.70 | -1801.61 | -1798.00 |
| Akaike Inf. Crit. | 3663.40 | 3635.23 | 3632.00 |
|  | |  |  |
| Note: ^*^p<0.1; ^**^p<0.05; ^***^p<0.01 | | | |
