# Supplementary material for: Sociocultural heterogeneity in a common pool resource dilemma
Source: PLoS One. 2019 Jan 17;14(1):e0210561. doi: 10.1371/journal.pone.0210561 (PMC6336341; doi:10.1371/journal.pone.0210561)
Supplement: S6 Text — (DOCX) [file pone.0210561.s008.docx]

**S6 Text. Principal Component Analysis for wealth**

PC1 of varimax-rotated Principal Component Analysis for construction of wealth index. It correlates with rather advanced, expensive and rare items. Factor loadings < 0.1 are not reported.

| Item | PC1 (Wealth) | PC2 | Rel. Frequency |
| --- | --- | --- | --- |
| Electricity | 0.83 |  | 0.37 |
| Fan | 0.76 |  | 0.18 |
| Sitting toilet | 0.14 | 0.39 | 0.23 |
| Radio | 0.12 | 0.20 | 0.81 |
| TV | 0.86 |  | 0.30 |
| Satellite dish | 0.47 | 0.15 | 0.04 |
| VCR/DVD | 0.85 |  | 0.26 |
| Pay-TV | 0.73 | 0.20 | 0.13 |
| Fridge | 0.75 | 0.16 | 0.19 |
| Smartphone | 0.43 |  | 0.27 |
| Cupboard | 0.17 | 0.40 | 0.80 |
| Chairs | 0.15 | 0.48 | 0.38 |
| Modern stove | 0.59 |  | 0.06 |
| Cement walls |  | 0.71 | 0.87 |
| Cement floor |  | 0.54 | 0.98 |
| Metal roof |  | 0.69 | 0.95 |
| Motorcycle |  |  | 0.03 |
| Car | 0.11 | -0.49 | 0.06 |
| SS loadings | 4.58 | 2.20 |  |
| Cumul. variance | 0.26 | 0.38 |  |
