# Supplementary material for: Sociocultural heterogeneity in a common pool resource dilemma
Source: PLoS One. 2019 Jan 17;14(1):e0210561. doi: 10.1371/journal.pone.0210561 (PMC6336341; doi:10.1371/journal.pone.0210561)
Supplement: S7 Text — (DOCX) [file pone.0210561.s009.docx]

**S7 Text. Additional Tobit panel regressions**

We re-estimate Model 2, Table 3 from the main text, with additional variables from the questionnaire-based survey that was administered after the experiments (see S4 Text for original questions), in order to see if these individual-level proxies for sustainable behaviour or concerns can explain extraction in the CPR experiment:

- Future worry (1 if worried about children having to use the same fishing grounds in the future, 0 if otherwise)
- Dragnet (1 if dragnet is main gear for fishing, 0 if other gear)
- Net (1 if dragnet or purse seine net is main gear for fishing, 0 if other gear)
- Dragnet destructive (1 if dragnet judged as most harmful fishing technique, 0 if other gear)

In the first column, we use dragnet as dummy (those fishers that self-reported to use the dragnet technique). In the second column, we use net in the wider sense as a dummy (those fishers that self-reported to use dragnet or purse seine net. Both nets can in principle be used for dragging).

We find that a proxy for concern about sustainability (worrying about the future of the fishing grounds) has no effect on experimental extraction, neither does using potentially destructive fishing techniques (dragnets), nor is there an interaction effect. Thus, fishers who use dragnets and are concerned about their destructive effects (i.e., those that might be constrained in their gear choice by external factors despite having environmental concern) do not show different CPR game behaviour from those who use dragnet but are not concerned. Note that estimates of village-level effects are not affected by inclusion of these additional variables, which shows that these individual-level variables cannot explain the village effect.

|  | |  | |
| --- | --- | --- | --- |
|  | *Dependent variable:* | | |
|  |  |  | |
|  | Extraction | | |
|  | (1) | | (2) |
| Heterogeneity | 0.41 (0.53) | 0.47 (0.54) | |
| CH | -1.47^***^ (0.41) | -1.53^***^ (0.44) | |
| Heterogeneity x CH | -1.31^*^ (0.67) | 1.40^*^ (0.72) | |
| Age | -0.002 (0.01) | -0.003 (0.02) | |
| Income | -0.0003 (0.03) | 0.002 (0.03) | |
| Wealth | 0.29 (0.18) | 0.25 (0.19) | |
| Household size | -0.08 (0.08) | -0.09 (0.09) | |
| Dragnet destructive | 0.08 (0.65) | -0.57 (0.96) | |
| Dragnet | -0.22 (1.36) |  | |
| Dragnet x Dragnet destructive | -0.63 (1.46) |  | |
| Net |  | -0.89 (1.03) | |
| Net x Dragnet destructive |  | 0.66 (1.13) | |
| Future worry | 0.28 (0.40) | 0.04 (0.38) | |
| Round | 0.08^***^ (0.03) | 0.08^***^ (0.03) | |
| Constant | 6.18^***^ (0.94) | 6.88^***^ (1.15) | |
|  | |  | |
| Left censored | 75 | 75 | |
| Right censored | 202 | 202 | |
| Observations (Subjects) | 944 (108) | 944 (108) | |
| Log Likelihood | -1,882.67 | -1,883.34 | |
| Akaike Inf. Crit. | 3,795.34 | 3,796.67 | |
|  | |  | |
| Note: ^*^p<0.1; ^**^p<0.05; ^***^p<0.01 | | | |
|  | | | |
