# Supplementary material for: Sociocultural heterogeneity in a common pool resource dilemma
Source: PLoS One. 2019 Jan 17;14(1):e0210561. doi: 10.1371/journal.pone.0210561 (PMC6336341; doi:10.1371/journal.pone.0210561)
Supplement: S8 Text — (DOCX) [file pone.0210561.s010.docx]

**S8 Text. Self-reported strategic goals**

Relative frequency of responses in the survey:

|  | earn as much as possible | earn more than the others | earn as much as the others | make others earn as much as possible | other |
| --- | --- | --- | --- | --- | --- |
| CH (N = 68) | 16.2 % | 45.6 % | 35.3 % | 1.5 % | 1.5 % |
| MA (N = 40) | 17.5 % | 45.0 % | 30.0 % | 5.0 % | 2.5 % |
| Total (N = 108) | 16.7 % | 45.4 % | 33.3 % | 2.8 % | 1.9 % |

Test for a village difference:

Χ^2^ = 1.51, df = 4, p = 0.82
